# Identification of Glucocorticoid Receptor Target Genes That Potentially Inhibit Collagen Synthesis in Human Dermal Fibroblasts

**DOI:** 10.3390/biom13060978

**Published:** 2023-06-11

**Authors:** Dabin Choi, Wesuk Kang, Soyoon Park, Bomin Son, Taesun Park

**Affiliations:** Department of Food and Nutrition, BK21 FOUR, Yonsei University, 50 Yonsei-ro, Seodaemun-gu, Seoul 120-749, Republic of Korea

**Keywords:** glucocorticoid, collagen, glucocorticoid receptor, dermal fibroblast

## Abstract

Over several decades, excess glucocorticoids (GCs) of endogenous or exogenous origin have been recognized to significantly inhibit collagen synthesis and accelerate skin aging. However, little is known regarding their molecular mechanisms. We hypothesized that the action of GCs on collagen production is at least partially through the glucocorticoid receptor (GR) and its target genes, and therefore aimed to identify GR target genes that potentially inhibit collagen synthesis in Hs68 human dermal fibroblasts. We first confirmed that dexamethasone, a synthetic GC, induced canonical GR signaling in dermal fibroblasts. We then collected 108 candidates for GR target genes reported in previous studies on GR target genes and verified that 17 genes were transcriptionally upregulated in dexamethasone-treated dermal fibroblasts. Subsequently, by individual knockdown of the 17 genes, we identified that six genes, AT-rich interaction domain 5B, FK506 binding protein 5, lysyl oxidase, methylenetetrahydrofolate dehydrogenase (NADP + dependent) 2, zinc finger protein 36, and zinc fingers and homeoboxes 3, are potentially involved in GC-mediated inhibition of collagen synthesis. The present study sheds light on the molecular mechanisms of GC-mediated skin aging and provides a basis for further research on the biological characteristics of individual GR target genes.

## 1. Introduction

Glucocorticoid hormones (GCs) mediate several metabolic actions, including regulation of glucose, protein, and fat metabolism, in response to various physical and psychological stresses [1,2]. This hormone exerts its actions through the intracellular glucocorticoid receptor (GR), a transcription factor that belongs to the nuclear receptor superfamily. GRs are typically located in the cytoplasm of various cell types and almost all tissues in mammals. After binding to GC hormones, GRs are phosphorylated and translocated to the nucleus, where they modulate gene transcription by interacting with specific sequences (GC-responsive elements, GREs) in the promoter region of several target genes (about 5% genome) [3,4,5]. In addition to the genomic pathway, GCs also demonstrate non-genomic actions. These constitute rapid effects that are not mediated by gene transcription. The non-genomic actions of glucocorticoids, frequently mediated by membrane-associated receptors, involve multiple signal transduction pathways, such as kinase activation. Depending on the cellular context and specific metabolic process, these actions can either complement or counteract the genomic effects [6,7,8,9]. Although several studies have already identified most GR target genes using multiple methods, including genome-wide association studies and chromatin immunoprecipitation [10,11,12,13,14,15], the contribution of each of these genes to specific GC-mediated metabolic processes is not fully understood.

In the human body, GCs are endogenously synthesized primarily in the adrenal cortex in response to various physical/psychological stresses and are released into the blood circulation. Furthermore, glucocorticoids can also be synthesized in other tissues/organs, including the skin. In fact, the skin functions as a unique site for glucocorticoid production, operating independently from the hypothalamic–pituitary–adrenal axis. This distinct function has led researchers to refer to the skin as a form of ‘peripheral brain’, illustrating its independent role in hormonal regulation [16,17,18]. In addition to endogenous synthesis, the human body can be directly exposed to GCs through the administration of topical agents that are highly effective in the treatment of inflammatory skin diseases, including atopic dermatitis [19,20,21,22]. However, until recently, GC-induced skin atrophy was considered one of the most prevalent side effects, with changes occurring in all skin compartments, including the epidermis and dermis, such as marked hypoplasia, increased fragility, dysfunctional skin barrier, or wrinkled skin [23,24,25,26]. The topical application of GC to healthy human skin causes skin atrophy characterized by decreased skin thickness and loss of elasticity with tearing [25,27]. Furthermore, a clinical study of participants without prior skin disease demonstrated that the barrier recovery rate (after the barrier disruption was implemented by tape stripping) decreased in parallel with the increased perceived psychological stress [28].

GC-induced epidermal atrophy and its underlying mechanisms have been relatively well established; recent studies have shown that FK506-binding protein 5 (FKBP5) and DNA damage-inducible transcript 4 (DDIT4), which are well-known GR target genes, contribute to epidermal atrophy; the knockdown of each gene results in considerable resistance to GC-induced epidermal atrophy [29,30]. On the other hand, in the dermis, it is well known that GC-induced reduction in collagen (the major structural protein with outstanding mechanical properties such as elasticity and resilience) causes dermal atrophy [31,32,33]; however, the molecular mechanism of the association between GC and collagen synthesis has not been extensively investigated yet. We hypothesized that some of the target genes directly regulated by GR in dermal fibroblasts could be partially related to collagen decrease; therefore, our objective was to identify the target genes involved in the inhibition of collagen synthesis induced by GC.

## 2. Materials and Methods

### 2.1. Cell Culture

Human dermal fibroblast Hs68 cells were obtained from the American Type Culture Collection (Manassas, VA, USA) and cultured in Dulbecco’s modified Eagle medium (DMEM; Gibco; Eggenstein, Germany) with 10% fetal bovine serum (FBS; Gibco) and 1% streptomycin–penicillin solution (Gibco) at 37 °C in a humidified incubator at 5% CO_2_. Mycoplasma was also removed using MycoZap Mycoplasma Elimination Reagent (Lonza; Gloucestershire, UK). The medium was changed every 2 days and the cells were detached with 0.25% trypsin-EDTA (Gibco) for subsequent experiments when they reached 80% confluence.

### 2.2. Measurement of Procollagen Type I C-peptide

Hs68 cells (4 × 10^4^ cells/well) were seeded in 24-well microplates and cultured for 24 h. The culture medium was discarded and each well was washed with phosphate-buffered saline (PBS; Welgene, Daegu, Republic of Korea). Then a serum-free medium with vehicle (dimethyl sulfoxide; DMSO) or 1 µM dexamethasone (Sigma-Aldrich, Seoul, Republic of Korea) was added to the culture plates and incubated for 48 h. The culture supernatant was collected and the levels of procollagen type I C-peptide were measured using a procollagen type I C-peptide enzyme-linked immunosorbent assay (ELISA) kit (Takara, Shiga, Japan). The optical density of each well at 590 nm was calculated using an Infinite M200 microplate reader (Tecan, Männedorf, Switzerland). Type I C-peptide procollagen levels were normalized to total protein concentrations and determined using the Bradford assay (Bio-Rad, Hercules, CA, USA).

### 2.3. Quantitative Real-Time Polymerase Chain Reaction (qPCR)

Hs68 cells (2 × 10^5^ cells/well) were seeded in 6-well microplates and incubated for 24 h. The cells were gently rinsed with PBS and serum-free medium with vehicle or 1 µM dexamethasone was added to each well. After incubation for 12 h, total RNA was isolated from the cells with Trizol (Invitrogen, Carlsbad, CA, USA) solution and reverse-transcribed into cDNA using SuperScript Ⅳ reverse transcriptase (Invitrogen). PCR amplification of cDNA was performed using 10 µL SYBR Green Supermix (Bio-Rad), 20 pmol primers, and 50 ng cDNA template on the CFX Real-Time System (Bio-Rad). All qPCR results were analyzed using the 2^(−ΔΔCt)^ method. The primer sequences are listed in Supplementary Table S1. All primers were validated by single-peak dissociation curves.

### 2.4. Next-Generation Sequencing (NGS)

NGS libraries were constructed from total RNA according to the NEBNext Ultra Ⅱ Directional RNA-Seq Kit protocol (BioLabs, Inc., New England, UK). For standard RNAseq, mRNA was isolated from total RNA and transcribed into cDNA. The cDNA fragments were generated through shearing and subsequently indexed using Illumina indexes 1–12. Thereafter, RNA-Seq was performed on the HiSeq X10 (paired-end 100 bp) sequencing platform (Illumina, San Diego, CA, USA). The abundance of the transcript is indicated in fragments per kilobase exon per million mapped fragments mapped (FPKM).

### 2.5. Small Interfering RNA (siRNA) Transfection

For siRNA screening, 4 × 10^4^ Hs68 cells were seeded in each well of 24-well microplates. After 24 h, the cells were transfected with non-targeting siRNA or specific siRNAs against upregulated genes by dexamethasone (final concentration 100 nM; Dharmacon, Lafayette, CO, USA) in the presence of Lipofectamine 3000 (Invitrogen), according to the manufacturer’s instructions, and further incubated for 48 h. The siRNA sequences are listed in Supplementary Table S2.

### 2.6. Human Phosphokinase Array

Hs68 cells were treated with vehicle or 1 µM dexamethasone and total protein was isolated using a specific buffer from the Human Phospho-Kinase Array (R&D Systems; Minneapolis, MN, USA) according to the manufacturer’s protocol. The protein concentration in the lysates was quantified using a bicinchoninic acid (BCA) Protein Assay Kit (R&D Systems) and 43 kinase phosphorylation sites were detected on phosphokinase array membranes. The array was rinsed to remove unbound proteins and incubated with biotinylated detection antibodies. Streptavidin–horseradish peroxidase (HRP, Sigma-Aldrich) and electrochemiluminescence detection reagents (Biomax, Seoul, Republic of Korea) were used, and the signals were recorded at each capture spot corresponding to the amount of phosphorylated protein bound using Light-capture (ATTO, Tokyo, Japan).

### 2.7. Western Blot

Hs68 cells (2 × 10^5^ cells/well) were seeded in 6-well microplates and cultured with vehicle or 1 µM dexamethasone. The cells were lysed by dissolving the cell pellets in the PRO-PREP protein extraction solution (iNtRON; Seoul, Republic of Korea). The whole cell lysates were centrifuged at 15,000× *g* and 4 °C for 20 min, and the supernatant was used for further analysis. The protein samples were denatured for 5 min at 95 °C, loaded onto 10% sodium dodecyl sulfate-polyacrylamide gel at 100 V for 1 h, and transferred onto a nitrocellulose membrane. The membrane was blocked with 5% bovine serum albumin (BSA; LPS solution, Daejeon, Republic of Korea) and then incubated for 16 h at 4 °C with diluted primary antibodies (1:1000; Cell Signaling Technology, Beverly, MA, USA). The HRP-conjugated IgG secondary antibody (Sigma-Aldrich) diluted at 1:5000 was used as the secondary antibody and the nitrocellulose membrane was incubated for 1 h at 25 °C. Protein bands were visualized using an electrochemiluminescence detection reagent (Biomax) and Light-capture (ATTO). For the assessment of collagen in culture medium, a 20 µL aliquot of each sample was loaded on the gel and subjected to electrophoresis under reduced denaturing conditions. The primary antibody employed was a rabbit polyclonal antibody against collagen I (1:1000; Abcam, Cambridge, MA, USA). To determine the collagen type I concentration in the culture medium, the density measurements of the final protein bands were normalized with the protein content of the cells in the corresponding well.

### 2.8. Reporter Gene Transfection and Dual Luciferase Assay

For DNA transient transfection experiments, 4 × 10^6^ Hs68 cells were seeded in each well of 24-well microplates and incubated until 70% confluency. The medium was replaced with serum/antibiotic-free medium and the cells were transiently transfected with 250 ng pGRE-luc and 25 ng pRL-TK plasmid with 0.75 µL Lipofectamine 3000, according to the manufacturer’s protocol. After 24 h, cells were washed with PBS, and serum/antibiotic-free medium with or without dexamethasone was added to each well. After incubation with dexamethasone for the indicated time, the cells were rinsed with cold PBS and lysed with passive lysis buffer (Promega; Southampton, UK). Firefly luciferase and *Renilla* luciferase activity levels were estimated using a luminometer (GloMax 20/20; Promega) and the Dual-Luciferase Reporter Assay System (Promega). The luciferase activity was normalized to that of *Renilla* luciferase and the values are shown as fold increases compared to the untreated control.

### 2.9. Statistical Analysis

The statistical significance of the differences between the groups was examined using SPSS25 software (Chicago, IL, USA) and all quantitative results are presented as mean ± standard error of the mean (SEM). The unpaired Student’s *t*-test was used to compare parameters between the two groups. For all tests, three significance levels, *p* < 0.05 (*), *p* < 0.01 (**), and *p* < 0.001 (***), were used.

## 3. Results

### 3.1. Dexamethasone Activated the Typical GR Signaling Pathway in Human Dermal Fibroblasts

**Figure 1:**
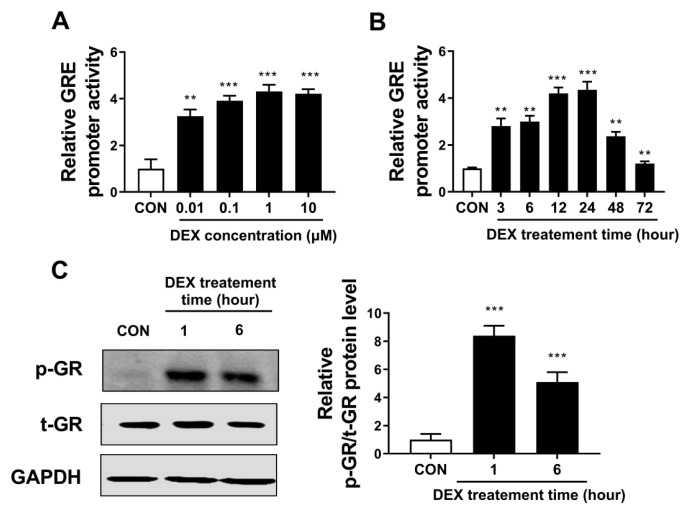
Dexamethasone activated the typical GR signaling pathway in human dermal fibroblasts. (**A**,**B**) Hs68 cells were transfected with the glucocorticoid response element firefly luciferase plasmid (pGRE-luc) and *Renilla* luciferase plasmid (pRL-TK). At 24 h after transfection, cells were treated with dimethyl sulfoxide (DMSO; CON) or various concentrations of dexamethasone (0.01–10 μM). Dual-luciferase assays were performed 24 h after treatment. (**B**) After transfection, Hs68 cells were treated with DMSO or dexamethasone (1 μM) and incubated for the indicated time, and dual-luciferase assays were performed. (**C**) Hs68 cells were treated with DMSO or dexamethasone for 1 and 6 h. Expression levels of phospho-GR (p-GR; S211), total-GR (t-GR), and glyceraldehyde 3-phosphate dehydrogenase (GAPDH) expression levels were measured by Western blotting. The data are represented as the mean ± standard error of the mean (SEM) of three replicates. ** *p* < 0.01, *** *p* < 0.001.

First, the activity of the GRE promoter and GR phosphorylation were evaluated to confirm whether dexamethasone (synthetic GC) activates the typical GR signaling pathway and subsequent activation of the GRE promoter. Treatment with dexamethasone for 24 h dramatically increased GRE promoter activity, and this effect appeared to be maximal at around 1 μM dexamethasone (Figure 1A). Based on this finding, all subsequent experiments were conducted at a concentration of 1 μM dexamethasone. Furthermore, we confirmed that dexamethasone (1 μM) substantially increased GRE promoter activity in a time-dependent manner, showing the peak elevation at 12–24 h (Figure 1B). Then we showed that dexamethasone successfully enhanced GR phosphorylation at S211 in human dermal fibroblasts prior to GRE activation (Figure 1C).

### 3.2. Dexamethasone Inhibited Collagen Synthesis in Human Dermal Fibroblasts

**Figure 2:**
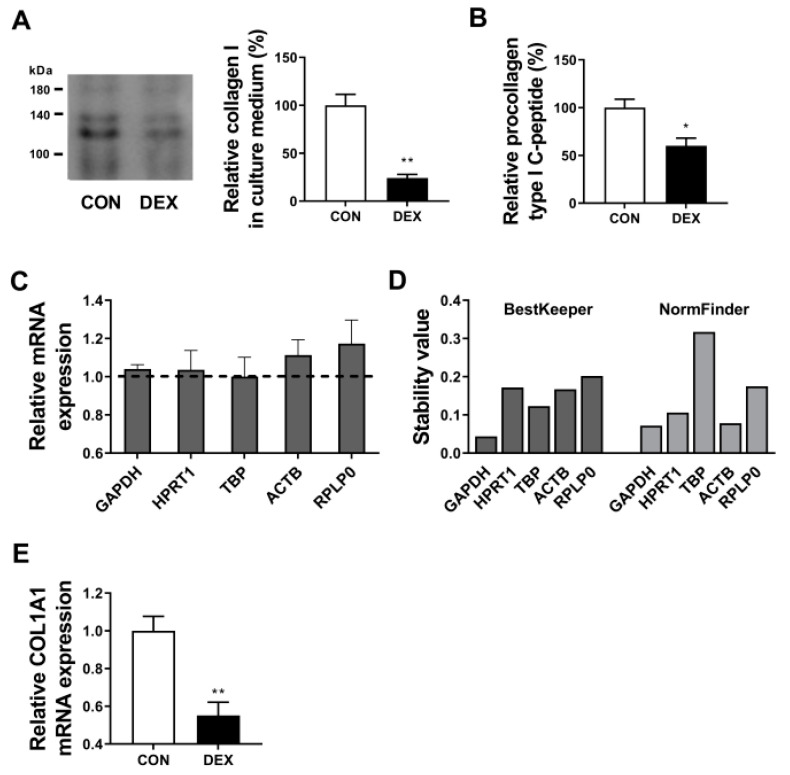
Dexamethasone inhibited collagen synthesis in human dermal fibroblasts. Hs68 cells were treated with vehicle (DMSO) or dexamethasone (1 μM) for 48 h, and (**A**) type I collagen (typically expressed as two isoforms around 120–140 kDa) was determined and (**B**) procollagen type I c-peptide concentrations in the culture medium were determined by Western blot and ELISA, respectively. (**C**) Relative mRNA expression of housekeeping genes including glyceraldehyde-3-phosphate dehydrogenase (GAPDH), hypoxanthine phosphoribosyltransferase 1 (HPRT1), TATA-box binding protein (TBP), actin beta (ACTB), and ribosomal protein lateral stalk subunit P0 (RPLP0) in 1 μM dexamethasone-treated hs68 cells were compared with the average relative expression of non-treated cells selected as a control and set to 1, which is represented by a dotted line. (**D**) Stability analysis of candidate reference gene expression analyzed by BestKeeper and NormFinder. Lower values indicate more stable gene expression. (**E**) The levels of collagen type I alpha 1 chain (*COL1A1*) mRNA expression in Hs68 cells were determined by RT-qPCR 24 h after dexamethasone administration (1 μM). The data are represented as the mean ± standard error of the mean (SEM) of three replicates. * *p* < 0.05, ** *p* < 0.01.

To confirm the inhibitory effect of GC on collagen synthesis, we measured the concentration of procollagen type I in control or dexamethasone-treated human dermal fibroblasts. Treatment with dexamethasone significantly reduced type I collagen synthesis in the culture medium, as measured by Western blot (Figure 2A). In line with the result of the Western blot assay, dexamethasone significantly decreased the levels of procollagen type I c-peptide, a biochemical marker for type I collagen synthesis that is removed from procollagen during collagen fiber formation in the extracellular process (Figure 2B). Before identifying changes in the collagen gene and screening the target gene that mediates this change under the influence of dexamethasone, it was necessary to establish a reliable reference gene in the current experimental conditions. We aimed to identify genes with stable expression, so genes with relative expression values close to 1 were considered ideal for comparison in cells exposed to dexamethasone. Based on this point, five widely used housekeeping genes, glyceraldehyde-3-phosphate dehydrogenase (GAPDH), hypoxanthine phosphoribosyltransferase 1 (HPRT1), TATA-box binding protein (TBP), actin beta (ACTB), and ribosomal protein lateral stalk subunit P0 (RPLP0), were selected and, of these, GAPDH, HRPT1, and TPB were identified as relatively invariable in dermal fibroblasts upon dexamethasone treatment (Figure 2C). To further confirm the stability of the expression of the reference genes, both BestKeeper and NormFinder software were used [34,35]. The results showed that the most stable gene (the lowest stability value) was GAPDH (Figure 2D). Therefore, GAPDH was chosen as the reference gene for all subsequent qPCR experiments to ensure accurate quantification of RNA expression. Next, we confirmed using qPCR that treatment with dexamethasone resulted in a reduction in the expression of the collagen type I alpha 1 chain gene, which is consistent with the result of the type I collagen protein (Figure 2E).

### 3.3. Identification of GR Target Genes with Considerably Increased Expression in Dexamethasone-Treated Human Dermal Fibroblasts

Following the workflow described in Supplementary Figure S1, 108 candidate GR target genes were obtained from previously published GR target gene studies [10,11,12,13,14,15]. NGS has been widely used to perform absolute quantification of the transcriptome, which is the total set of transcripts present in a particular type of cell [36]. The expression levels of these 108 genes in human dermal fibroblasts were quantified using NGS and determined as FPKM values (Supplementary Table S3). Among these genes, we focused on the 89 genes with expression levels greater than 0.1 FPKM, which is generally considered a meaningful expression level according to previous studies [37,38].

**Figure 3:**
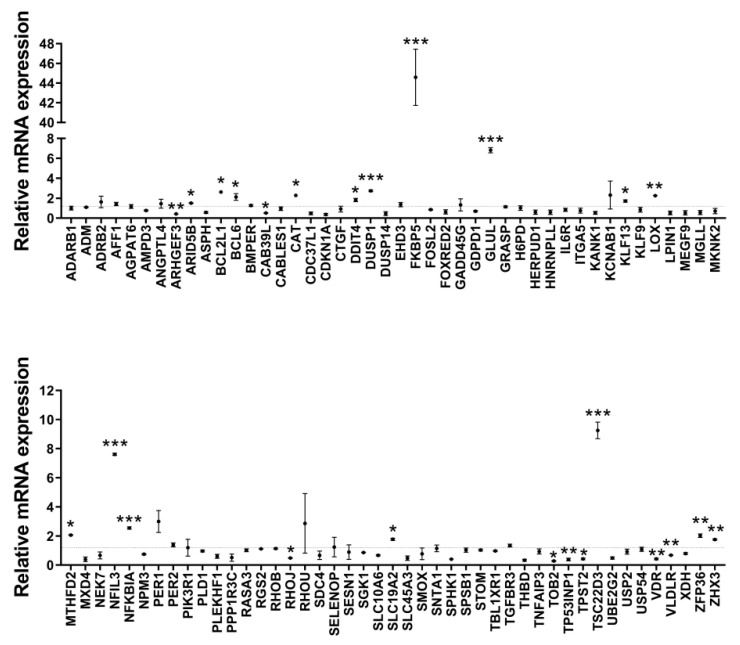
Identification of glucocorticoid receptor (GR) target genes with considerably increased expression in dexamethasone-treated human dermal fibroblasts. A and B, Hs68 cells were treated with dimethyl sulfoxide (DMSO) or 1 µM dexamethasone for 12 h. The fold changes in the 89 GR target genes after dexamethasone treatment were determined by RT-qPCR. Definitions of abbreviations are given in Supplementary Table S1. The data are represented as the mean ± standard error of the mean (SEM) of three replicates. * *p* < 0.05, ** *p* < 0.01, *** *p* < 0.001.

To examine whether dexamethasone stimulated the transcription of these genes in human dermal fibroblasts, we validated their expression by measuring mRNA levels using quantitative real-time PCR. Unexpectedly, dexamethasone significantly increased the mRNA levels of only 17 genes in human dermal fibroblasts, AT-rich interaction domain 5B (*ARID5B*), BCL2-like 1 (*BCL2L1*), BCL6 transcription repressor (*BCL6*), catalase (*CAT*), *DDIT4*, dual-specificity phosphatase 1 (*DUSP1*), *FKBP5*, glutamate-ammonia ligase (*GLUL*), kruppel-like factor 13 (*KLF13*), lysyl oxidase (*LOX*), methylenetetrahydrofolate dehydrogenase (NADP+ dependent) 2, methenyltetrahydrofolate cyclohydrolase (*MTHFD2*), nuclear factor, interleukin 3 regulated (*NFIL3*), NFKB inhibitor alpha (*NFKBIA*), solute carrier family 19 member 2 (*SLC19A2*), TSC22 domain family member 3 (*TSC22D3*), ZFP36 ring finger protein (*ZFP36*), and zinc fingers and homeoboxes 3 (*ZHX3*) (Figure 3, Table 1).

### 3.4. Dexamethasone-Induced Inhibition of Collagen Synthesis was Attenuated by the Knockdown of Six GR Target Genes

**Figure 4:**
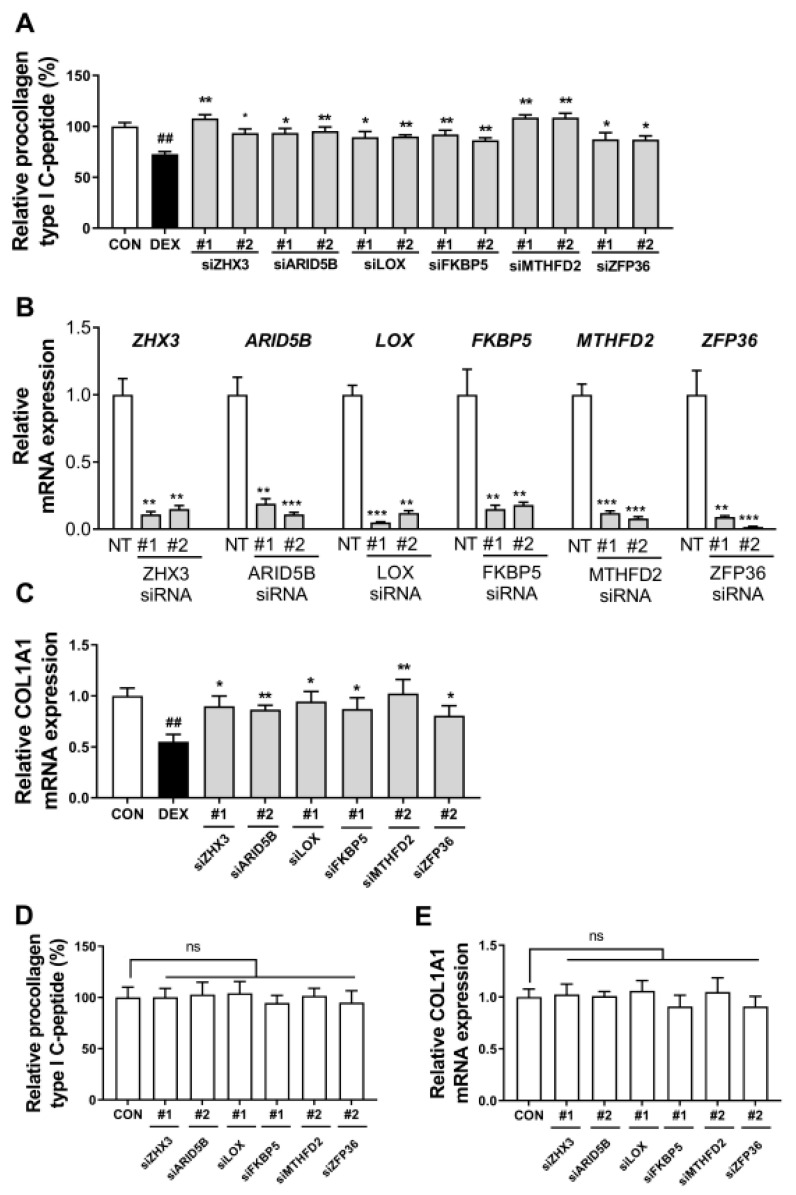
Dexamethasone-induced inhibition of collagen synthesis was attenuated by the knockdown of six glucocorticoid receptor target genes. (**A**) Hs68 cells were transfected with 100 nM non-targeting siRNA or two different small interfering RNA (siRNAs; 100 nM; #1, #2) each targeting zinc fingers and homeoboxes 3 (siZHX3), AT-rich interaction domain 5 B (siARID5B), lysyl oxidase (siLOX), FK506-binding protein 5 (siFKBP5), methylenetetrahydrofolate dehydrogenase (NADP+ dependent) 2, methenyltetrahydrofolate cyclohydrolase (siMTHFD2), and ZFP36 ring finger protein (siZFP36) for 48 h and then treated with 1 µM dexamethasone (DEX) for another 48 h. The relative procollagen type I c-peptide content in the cell culture medium was determined by enzyme-linked immunosorbent assay (ELISA). (**B**) Hs68 cells were transfected with 100 nM non-targeting siRNA (NT) or two different siRNAs (100 nM; #1, #2) each targeting indicated gene (100 nM) for 48 h. Then, *ARID5B*, *FKBP5*, *LOX*, *MTHFD2*, *ZFP36*, and *ZHX3* mRNA levels were examined using RT-qPCR. (**C**) Hs68 cells were transfected with the indicated siRNA (100 nM) for 48 h and then treated with 1 µM dexamethasone for another 24 h. The collagen type I alpha 1 chain (*COL1A1*) mRNA expression level in Hs68 cells was determined using RT-qPCR. (**D**,**E**) Hs68 cells were transfected with 100 nM non-targeting siRNA (CON) or siRNA targeting the indicated gene for 48 h, followed by treatment with dexamethasone vehicle (DMSO) for another 48 h, without dexamethasone treatment. Relative procollagen type I c-peptide in the culture medium and *COL1A1* mRNA expression levels in hs68 cells were determined by ELISA and RT-qPCR, respectively. The data are represented as the mean ± standard error of the mean (SEM) of three replicates. * *p* < 0.05, ** *p* < 0.01, *** *p* < 0.001 (vs. NT or DEX); ## *p* < 0.01 (vs. CON); ns, not significant.

Furthermore, we hypothesized that the inhibitory effect of dexamethasone on collagen synthesis is mediated by some of the 17 GR target genes that are upregulated by dexamethasone in dermal fibroblasts. We used a siRNA screening approach (the knockdown of each gene individually) to select the gene directly involved in the inhibition of collagen synthesis induced by dexamethasone. Consequently, the knockdown of *ARID5B*, *FKBP5*, *LOX*, *MTHFD2*, *ZFP36*, or *ZHX3* markedly attenuated the inhibition of dexamethasone-induced collagen synthesis (Figure 4A). This effect was not observed after the knockdown of 11 other GR target genes (Supplementary Figure S2). Furthermore, we validated the knockdown efficiency of the six siRNAs (siARID5B, siFKBP5, siLOX, siMTHFD2, siZFP36, and siZHX3) by detecting the expression level using RT-qPCR and confirmed that the expression decreased by over 80% in each Hs68 cell transfected with siRNA #1 or #2 (Figure 4B). In line with the collagen synthesis data, the knockdown of *ARID5B*, *FKBP5*, *LOX*, *MTHFD2*, *ZFP36*, or *ZHX3* using representative siRNA reversed the reduction in *COL1A1* mRNA expression in dermal fibroblasts exposed to dexamethasone (Figure 4C).

While knockdown of each gene significantly increased procollagen type I c-peptide in the presence of dexamethasone, we observed that there was no significant effect on collagen production in cells transfected with the same siRNA but not treated with dexamethasone (Figure 4D). Similarly, in Figure 4E, we compared the effects of different siRNA treatments on *COL1A1* mRNA expression in dexamethasone-untreated cells. Consistent with the results in Fig 4D, we found that there was no significant effect on collagen production in cells transfected with the same siRNA but not treated with dexamethasone. Taken together, these results indicate that the knockdown of each gene does not have a direct effect on collagen production but plays a role in mitigating the reduction of collagen production by dexamethasone.

### 3.5. Dexamethasone Modulated Collagen-Related Protein Kinase Signaling Pathways with the Possible Involvement of GR Target Genes in Human Dermal Fibroblasts

**Figure 5:**
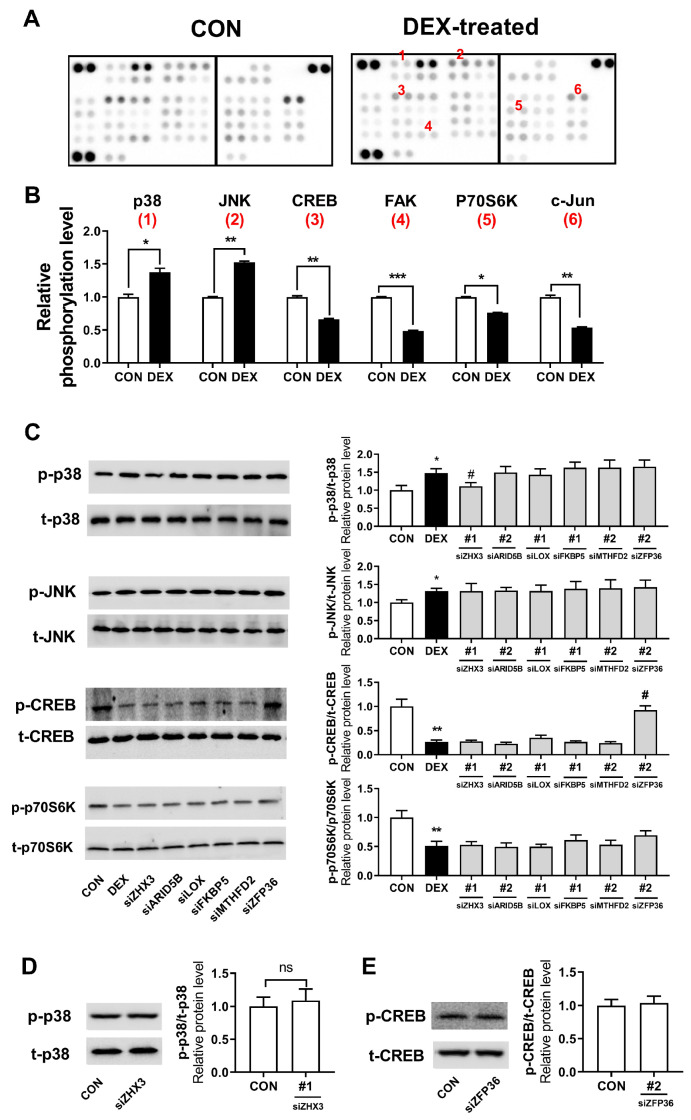
Dexamethasone modulated collagen-related protein kinase signaling pathways with the possible involvement of GR target genes in human dermal fibroblasts. (**A**,**B**) Whole-cell lysates were prepared from Hs68 cells treated with dimethyl sulfoxide (DMSO; CON) or 1 μM dexamethasone (DEX) for 24 h. Protein phosphorylation was analyzed using a phosphokinase array. The bar graph shows the changes in protein phosphorylation in dexamethasone-treated cells compared with those in DMSO-treated cells. (**C**) Hs68 cells were pretreated with non-targeting small interfering RNA (siRNA) or siRNAs targeting *ARID5B*, *FKBP5*, *LOX*, *MTHFD2*, *ZFP36*, or *ZHX3* for 48 h, then treated with dexamethasone for further 24 h. The protein levels of phospho-p38, total p38 (t-38), phospho-c-Jun N-terminal kinase (JNK), total JNK (t-JNK), Phospho-cAMP response element-binding protein (CREB), total CREB (t-CREB), phospho-p70 S6 kinase (p70S6K), and total p70S6K (t-p70S6K) were determined using immunoblotting. (**D**,**E**) Hs68 cells were pretreated with non-targeting siRNA (CON) or siRNAs targeting ZFP36 or ZHX3 for 48 h, followed by treatment with dexamethasone vehicle (DMSO) for another 24 h. The protein levels of phospho-p38, t-38, Phospho-CREB, and t-CREB were determined using immunoblotting. The data are represented as the mean ± standard error of the mean (SEM) of two (**A**,**B**) or three (**C**,**D**,**E**) replicates. * *p* < 0.05, ** *p* < 0.01, *** *p* < 0.001 (vs. CON); # *p* < 0.05 (vs. DEX); ns, not significant.

It is widely accepted that various kinases play a role in regulating collagen metabolism. We hypothesized that certain GR target genes, which modulate collagen synthesis in human dermal fibroblasts, participate in regulating these kinases and therefore control collagen synthesis. First, to explore potential alterations in cellular kinase signaling, we used a phosphokinase array consisting of 43 different kinases and examined their response to dexamethasone. Among the kinases examined, p38, c-Jun N-terminal kinase (JNK), cAMP response element binding protein (CREB), focal adhesion kinase (FAK), p70 S6 kinase (p70S6K), and c-Jun underwent significantly altered phosphorylation in dexamethasone-treated human dermal fibroblasts compared to phosphorylation in vehicle treated cells (Figure 5A,B). In this study, we narrowed down four candidate kinases (CREB, p38, p70S6K, and JNK), which are likely associated with collagen synthesis based on previous findings [39,40,41,42]. To evaluate this possibility that six GR target genes (*ARID5B*, *FKBP5*, *LOX*, *MTHFD2*, *ZFP36*, and *ZHX3*) may contribute to altered phosphorylation of these kinases, we tested the effect of the knockdown of these genes on CREB, p38, p70S6K, and JNK phosphorylation in the presence of dexamethasone. We observed that *ZHX3* knockdown selectively inhibited dexamethasone-induced p38 phosphorylation. Additionally, *ZFP36* knockdown substantially blocked the dexamethasone-mediated decrease in phosphorylation of CREB. The suppression of *ARID5B, FKBP5*, *LOX*, and *MTHFD2* did not influence the phosphorylation of CREB, p38, p70S6K, and JNK, respectively (Figure 5C). It remains uncertain whether the observed changes in phosphorylation of p-p38 upon ZHX3 knockdown and of p-CREB upon ZFP36 knockdown were caused by the dexamethasone blocking effects or by dexamethasone-independent off-target effects of the siRNA. To investigate this, we performed additional experiments under dexamethasone-untreated conditions and demonstrated that ZHX3 knockdown did not alter p-p38 phosphorylation levels in Hs68 cells without dexamethasone treatment (Figure 5D). Similarly, ZFP36 knockdown did not result in changes in p-CREB levels in dexamethasone-untreated Hs68 cells (Figure 5E). Thus, the observed alterations in phosphorylation following ZHX3 and ZFP36 knockdown seem to result in reduced dexamethasone efficacy, rather than being attributed to off-target effects.

Considering that ZFP36 and ZHX3 modulate collagen synthesis possibly through different mechanisms, we sought to explore the possibility of a synergistic effect when both genes are knocked down. Our results showed that the knockdown of either ZFP36 or ZHX3 led to a significant recovery of collagen synthesis in dexamethasone-treated dermal fibroblasts, but simultaneous knockdown of both ZFP36 and ZHX3 did not further enhance collagen synthesis compared to the individual knockdowns (Supplementary Figure S3). It appears that, if silencing of only one of these genes is sufficient to restore collagen synthesis to the levels observed in dexamethasone-untreated control, no synergistic effect beyond this level will occur.

**Figure 6:**
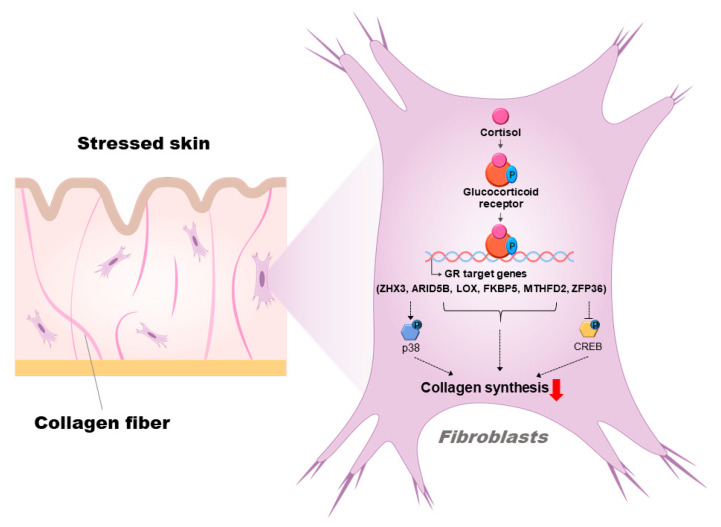
A schematic of how glucocorticoid receptor (GR) and its target genes can inhibit collagen synthesis in human dermal fibroblasts.

Taken together, these results indicated that GCs inhibit collagen synthesis, with the potential involvement of *ARID5B*, *FKBP5*, *LOX*, *MTHFD2*, *ZFP36*, and *ZHX3* in human dermal fibroblasts (Figure 6).

## 4. Discussion

In the current study, 1 μM of dexamethasone was used as an experimentally optimized concentration which fully activates the stress response. Interestingly, this level appears to correspond to the physiologically achievable level under the severe stressful conditions that can be encountered in daily life: the blood level of glucocorticoids in adults experiencing extreme psychological stress has been found to increase to around 1 μM [43,44,45]. For example, a clinical case observation study by Walvekar et al. revealed that the average level of blood cortisol in a group of individuals complaining of job-related stress was 1.13 μM (n = 41) but it was only 0.39 μM (n = 67) in an unstressed control group [43].

In addition to applications for controlling psychological-stress-associated collagen loss, our findings may have important implications for glucocorticoid therapeutics. In the past, some side effects were considered an unavoidable trade-off for the beneficial outcomes of topical glucocorticoid therapy but strategies to minimize side effects while maintaining the desired efficacy of glucocorticoids have recently been proposed. For example, a recent study demonstrated that a phosphoinositide 3-kinase inhibitor suppressed GR translocation and alleviated the glucocorticoid-induced collagen decrease while maintaining the anti-inflammatory effects of GCs [46]. Furthermore, in our previous study, we found that β-ionone attenuated the dexamethasone-induced suppression of collagen, while the expression of pro-inflammatory genes, which were decreased by dexamethasone, remained unchanged with β-ionone treatment [32]. Building on these findings, our research could provide the basis for developing a strategy that selectively targets genes involved in collagen reduction without affecting other beneficial pathways. This approach could help preserve the beneficial effects of glucocorticoids while minimizing the risks associated with their use in topical applications.

To broadly profile differentially expressed mRNAs between groups, several high-throughput technologies, such as microarray-based platforms, are commonly utilized. They cover almost the entire gene repertoire and can be effective and economical in screening specific treatment-induced gene regulation for further validation using quantitative PCR, which is the standard for mRNA evaluation. However, it has been increasingly suggested that these data are quite unreliable and often disagree with PCR data, which can result in the erroneous exclusion of several promising genes [47,48,49,50,51]. To avoid this possibility, we first selected promising GR target genes based on multiple studies that employed qualitative methods (such as chromatin immunoprecipitation) and then individually evaluated them in human dermal fibroblasts using PCR.

At the cellular level, both natural and synthetic GCs transduce their actions by binding to GR. The GC–GR complex binds to a target sequence (GRE) found in several cellular gene promoters; a bioinformatic search against the human transcriptome indicated that at least 5% of human genes harbor GRE motifs [5,52,53]. However, the presence of GREs in the gene promoter is not enough for GR-modulated gene expression and the set of target genes is highly context-specific. Indeed, the family of GR-responsive genes is vastly different from one cell type to another. Although cell-specific transcriptional regulatory mechanisms are largely unknown, they may be partially associated with differences in baseline expression of each gene and chromatin accessibility of target genes, thus modulating the availability of gene sequences to interact with the transcription machinery [13,54]. In the present study, we identified for the first time 17 functionally responsive GR target genes in human dermal fibroblasts.

Notably, even a single gene knockdown among six genes (*ARID5B*, *FKBP5*, *LOX*, *MTHFD2*, *ZFP36*, and *ZHX3*) in dermal fibroblasts effectively reversed the reduction of collagen induced by dexamethasone. It is possible to hypothesize that stress-induced collagen inhibition operates in a highly complicated and coordinated manner and there is no central gene. Furthermore, we selected 89 GR target genes with expression levels greater than 0.1 FPKM. They could also be divided into two groups depending on their expression levels: high expression group (45 genes; FPKM > 3) and low expression group (44 genes; FPKM < 3). All six genes are classified into the high expression group, implying that strong expression can provide clues to identify the GR target genes responsible for the distinct action of glucocorticoids.

One focus of our future work will be to clarify how these GR target genes (*ARID5B*, *FKBP5*, *LOX*, *MTHFD2*, *ZFP36*, and *ZHX3*) could suppress collagen synthesis. It should be noted that the effects of GC target genes are highly associated with various intracellular protein phosphokinases [55,56,57,58]. ZHX3 is a member of the ZHX protein family, which includes ZHX1 and ZHX2; these proteins contain two zinc-finger motifs and four or five homeodomain motifs. Several studies have reported that ZHX proteins are ubiquitously expressed in cells and function as kinase activators, resulting in various physiological events [59,60,61]. For example, ZHX2 stimulates mitogen-activated protein kinase (MAPK) and drives cell growth and migration in the renal cell carcinoma (RCC) cell line [60]. ZFP36, also known as tristetraprolin, is an RNA-binding protein that plays a critical role in modulating protein kinases through the destabilization of mRNA for a wide array of upstream regulators (such as cytokines and growth factors) by binding to their AU-rich elements (AREs) [62,63,64]. In the present study, as a pioneering attempt, we found that *ZHX3* knockdown reversed the dexamethasone-induced upregulation of p38 phosphorylation, which is a candidate kinase for inhibiting collagen synthesis in human fibroblasts [39]. We also verified that *ZFP36* knockdown reversed the dexamethasone-mediated downregulation of phosphorylation of CREB, a well-known transcriptional factor of collagen gene [40]. More research is needed to determine the contributions of these GR target genes to the regulation of specific protein phosphokinases, including p38 and CREB.

The classical effects of ligand-bound GR signaling are exerted by direct high-affinity binding of GR to GREs found in the promoter regions of GC target genes. However, the mechanisms of action of GC-bound GR are quite complex, including the ability to interact with other transcription factors such as nuclear factor kappa B (NF-κB) and activating protein-1 (AP-1), which participate in additional layers of transcriptional regulation. Understanding the comprehensive nature of GC action requires consideration of not only the set of genes bound and activated by GR (canonical GR signaling) but also other transcription factors that could interact with GR (non-canonical GR signaling). In addition to their genomic activity, it is worth noting that glucocorticoids also exert rapid, non-genomic actions involving membrane-associated receptors and signaling pathways, leading to diverse physiological responses in target cells [7,65]. Further studies are needed to determine the diverse actions of GR in human dermal fibroblasts.

In our current study, we explored the genomic mechanisms underlying glucocorticoid-induced collagen reduction. However, skin aging, characterized by the loss of extracellular matrix (ECM) components, involves more than just collagen reduction. The ECM comprises various structural proteins, including collagen, fibronectin, and elastin. Therefore, the role of glucocorticoid target genes in the context of these proteins should be further examined in the future to better understand skin aging profoundly. Intriguingly, it has been reported that responses to glucocorticoids vary among ECM proteins. For instance, while collagen is known to decrease upon exposure to glucocorticoids, some reports suggest that fibronectin actually increases in the presence of glucocorticoids [66,67,68]. Investigating why such contrasting trends exist among the key components of ECM in the view of GR target genes could provide comprehensive insights into the role of glucocorticoids in skin aging.

Furthermore, the GR target genes identified in this study were not previously known to directly regulate collagen production. Importantly, several pathological conditions, including fibrotic diseases and certain types of cancer, typically involve upregulated glucocorticoid levels [69,70,71,72]. Both conditions are characterized by aberrant collagen synthesis: fibrotic diseases involve excessive collagen accumulation, leading to tissue stiffening, while many forms of cancer exhibit increased collagen synthesis, contributing to tumor progression [73,74,75,76]. Our findings are significant not only because they enhance our understanding of skin aging, but also because they could provide potential insights for interventions in conditions such as fibrotic diseases and cancer.

In summary, our study describes the identification of dexamethasone-activated GR target genes and suggests that six GR target genes (*ARID5B*, *FKBP5*, *LOX*, *MTHFD2*, *ZFP36*, and *ZHX3*) are directly involved in GC-induced inhibition of collagen synthesis in human dermal fibroblasts. These findings contribute to a deep understanding of the molecular mechanism of GC-mediated inhibition of collagen synthesis and consequent skin aging.

## Figures and Tables

**Table 1:** Upregulation of mRNA abundance of the glucocorticoid receptor (GR) target genes in dexamethasone (DEX)-treated human dermal fibroblasts.

Gene Name	Description	Fold Change (DEX/Control)	*p* Value
ARID5B	AT-rich interaction domain 5B	1.50	*
BCL2L1	BCL2 like 1	2.62	*
BCL6	BCL6 transcription repressor	2.12	*
CAT	Catalase	2.28	*
DDIT4	DNA damage inducible transcript 4	1.82	*
DUSP1	Dual specificity phosphatase 1	2.74	***
FKBP5	FK506 binding protein 5	44.58	***
GLUL	Glutamate-ammonia ligase	6.81	***
KLF13	Kruppel like factor 13	1.72	*
LOX	Lysyl oxidase	2.25	**
MTHFD2	Methylenetetrahydrofolate dehydrogenase (NADP+ dependent)	2.06	*
NFIL3	Nuclear factor, interleukin 3 regulated	7.61	**
NFKBIA	NFKB inhibitor alpha	2.55	***
SLC19A2	Solute carrier family 19 member 2	1.77	*
TSC22D3	TSC22 domain family member 3	9.25	***
ZFP36	ZFP36 ring finger protein	2.02	**
ZHX3	Zinc fingers and homeoboxes 3	1.75	**

## Data Availability

The data that support the findings of this study are available from the corresponding author upon reasonable request.

## Supplementary Materials

The following supporting information can be downloaded at: https://www.mdpi.com/article/10.3390/biom13060978/s1, Figure S1: The schematic workflow of the small interfering RNA (siRNA) screening for the identification of glucocorticoid receptor (GR) target genes involved in collagen synthesis; Figure S2: The inhibition of dexamethasone-induced collagen synthesis is not affected by the knockdown of 11 glucocorticoid receptor (GR) target genes; Figure S3: ZHX3 and ZFP36 knockdown show no synergistic effects on collagen synthesis; Table S1: Primer sequences for RT-qPCR; Table S2: Sequences of small interfering RNAs (siRNAs) targeting 17 glucocorticoid receptor (GR) target genes and non-targeting siRNA; Table S3: Fragments per kilobase exon per million fragments mapped (FPKM) values of 108 glucocorticoid receptor (GR) target genes in human dermal fibroblasts.
