# Supplementary material for: Identification of Glucocorticoid Receptor Target Genes That Potentially Inhibit Collagen Synthesis in Human Dermal Fibroblasts
Source: Biomolecules. 2023 Jun 11;13(6):978. doi: 10.3390/biom13060978 (PMC10296022; doi:10.3390/biom13060978)
Supplement: Supplementary file 1 [file biomolecules-13-00978-s001.zip › Supplementary figure.pdf]

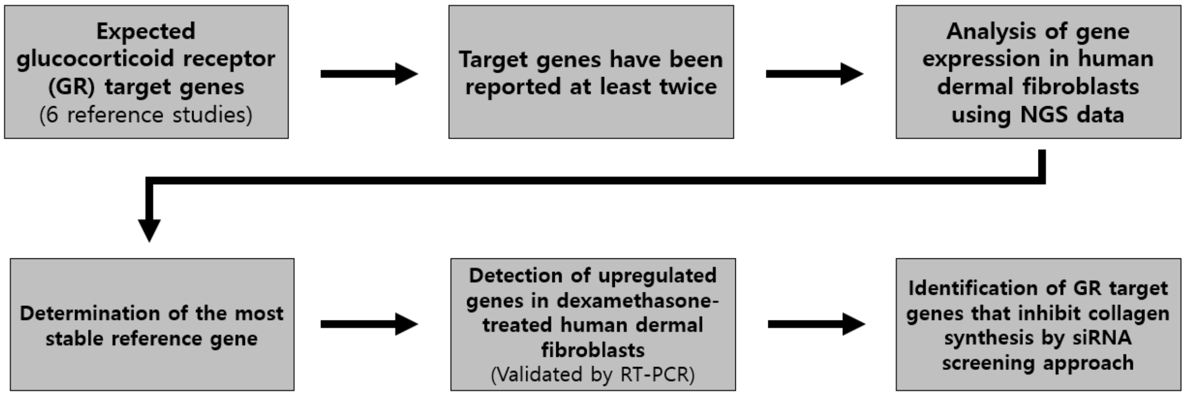

Supplementary Figure S1. The schematic workflow of the small interfering RNA (siRNA) screening for the identification of glucocorticoid receptor (GR) target genes involved in collagen synthesis. GR target genes were obtained from six independent sources and the genes reported at least twice were selected. Then we retained only the transcripts characterized by >0.1 fragments per kilobase exon per million fragments mapped (FPKM) according to next-generation sequencing (NGS) data. Subsequently, the identification of genes and collagen synthesis-inhibiting genes were validated using RT-qPCR and siRNA screening, respectively.

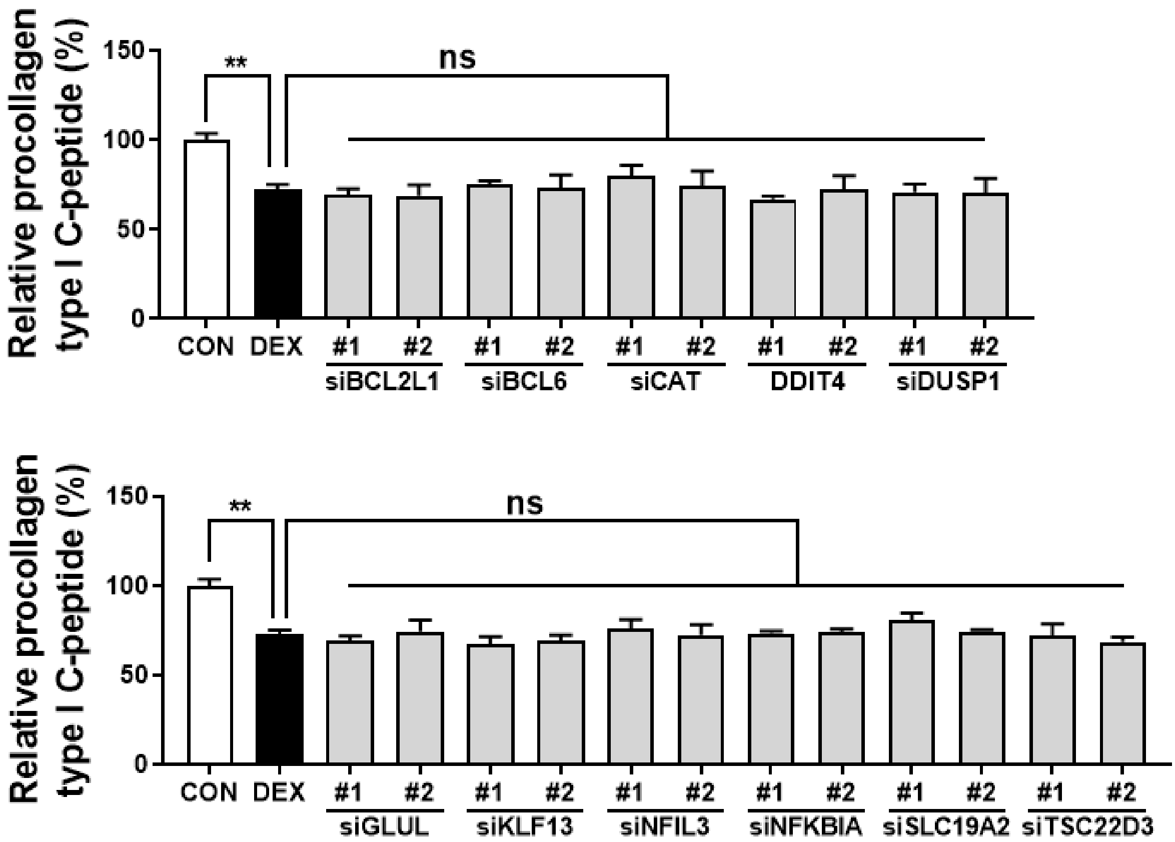

Supplementary Figure S2. The inhibition of dexamethasone-induced collagen synthesis is not affected by the knockdown of 11 glucocorticoid receptor (GR) target genes. Hs68 cells were transfected with 100 nM non-targeting siRNA (NT) or two different small interfering RNA (siRNAs; 100 nM; #1, #2) each targeting indicated gene for 48 h and then treated with 1 μM dexamethasone (DEX) for another 48 h. The relative procollagen in the cell culture medium was determined by ELISA. The data are represented as the mean ± standard error of the mean (SEM) of three replicates. ns>0.05, **P<0.01

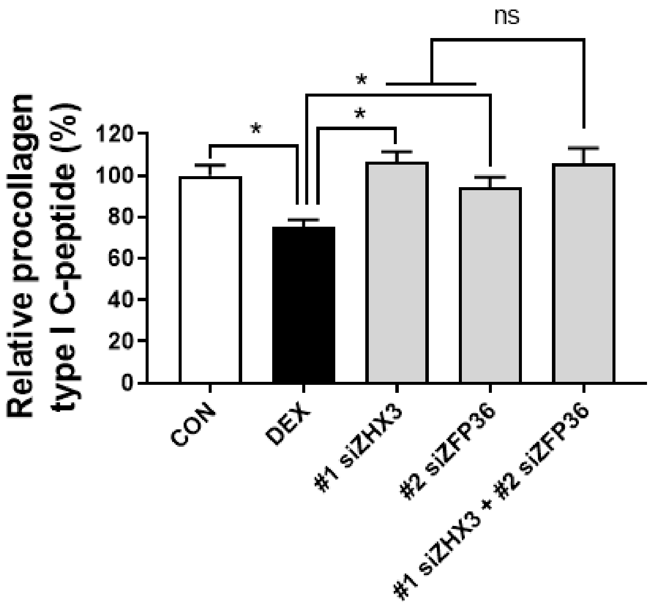

Supplementary Figure S3. ZHX3 and ZFP36 knockdown show no synergistic effects on collagen synthesis. Hs68 cells were transfected with either 100 nM non-targeting siRNA or specifically targeting zinc fingers and homeoboxes 3 (siZHX3), ZFP36 ring finger protein (siZFP36), or both siZHX3 and siZFP36 for 48 h. Following transfection, the cells were treated with 1 μM dexamethasone (DEX) for another 48 h. The relative procollagen type I c-peptide content in the cell culture medium was determined by enzyme-linked immunosorbent assay (ELISA). The data are represented as the mean ± standard error of the mean (SEM) of three replicates. *$P<0.05$; ns, not significant.
