# Supplementary material for: Identification of Glucocorticoid Receptor Target Genes That Potentially Inhibit Collagen Synthesis in Human Dermal Fibroblasts
Source: Biomolecules. 2023 Jun 11;13(6):978. doi: 10.3390/biom13060978 (PMC10296022; doi:10.3390/biom13060978)
Supplement: Supplementary file 1 [file biomolecules-13-00978-s001.zip › Supplementary Table.pdf]

Supplementary Table S1. Primer sequences for RT-qPCR.

Gene name	Description	Forward primer (5'→ 3')	Reverse primer (5'→ 3')
ADARB1	adenosine deaminase RNA specific B1	AAACTCCTGACAAGGCGGAG	GGGGTGGGAATGGTGGTAAG
ADM	adrenomedullin	CTTGCCACTTCGGGCTTCTC	CGACGGAAACCAGCTTCATCC
ADRB2	adrenoceptor beta 2	AAGCGGCTTCTTCAGAGCAC	TCCTGCACACTCAGCTTGTC
AFF1	AF4/FMR2 family member 1	AAGGCGCTCATGGACGGAAG	TCATTGGAGTAGGTCTGCTTGAC
AGPAT6	glycerol-3-phosphate acyltransferase 4	CTCCCCCAAGTCCCAGCTTTG	AGGCACCCTTTCTTAGCAGTG
AMPD3	adenosine monophosphate deaminase 3	GTCTCGCGCTGGGAAAAGTTG	AGCCGGACTTGCTCATCCAC
ANGPTL 4	angiopoietin like 4	GTACCCTTCTCCACTTGGGAC	AAACCACCAGCCTCCAGAGAG
ARHGEF 3	Rho guanine nucleotide exchange factor 3	CAGCTGTAGAGGAATGGAAGAA AAC	CCAGGCTTTAGGAGAGGGAAAC
ARID5B	AT-rich interaction domain 5B	GAGATGGAGCCCAACTCACTC	CTGGTCCTCTCTTCCCACAAC
ASPH	aspartate beta-hydroxylase	CCCAGCGTAAGAATGCCAAG	CTGAGAGTCCGCCTTTCCTC
BCL2L1	BCL2 like 1	CGTAAGACCCACAAAGAAACCA G	TGAGTCTCGTCTCTGGTTAGTG
BCL6	BCL6 transcription repressor	CAGCCTAGGTATTCCAGAAGTG	TGCAAAAGAAAGCTGAAGACAC
BMPER	BMP binding endothelial regulato	CTGTCCCCAGCACCTTAGTC	CAGCTCCCAAAAGGGAGGTC
CAB39L	calcium binding protein 39 like	CGAGAAAGAACCCCCAACAGAA G	TGAGGAGCATAAACAGGATATGA GG
CABLES1	Cdk5 and Abl enzyme substrate 1	TCATTGGTCTGGAAGGTGTGGAG	GAACTGGGAGAAAGAGGAGGTG
CAT	catalase	TGCAGTCCGGATCTCACTTG	ATGAGCGGGTTACACGGATG
CDC37L1	cell division cycle 37 like 1	GTCGATGGGATGATAGCCAG	TCAGCTTCCAGGTGAAAACAC
CDKN1A	cyclin dependent kinase inhibitor 1A	GAGCTCTGGCATAGAAGAGGCTG	TTCTGACATGGCGCCTGAAAAC
CTGF	connective tissue growth factor	CAGCATGGACGTTCGTCTG	AACCACGGTTTGGTCCTTGG
DDIT4	DNA damage inducible transcript 4	TCCGGCACTCTGAGTTCATC	ACGAGAAGCGGTCCCAAAG
DUSP1	dual specificity phosphatase 1	GAGCCCCATTACGACCTCTC	GAGCCCCTCCCAGAGTTATTG
DUSP14	dual specificity phosphatase 14	ACGGAGCCCTAACCGCAAC	ATCCAGAAAGTCCTAAGTGAGCA G
EHD3	EH domain containing 3	GCGGAGCCGAAGCATC	AAAGTTGAACCTGCAAGGAAACT G
FKBP5	FK506 binding protein 5	CATCAAGGCATGGGACATTGG	TCTACTGTTGCTCCTTCGTTTG
FOSL2	FOS like 2, AP-1 transcription factor subunit	CTACTTGAGCCCCACGAGC	GGCGGCTCGGATACTTGAC
FOXRED2	FAD dependent oxidoreductase domain containing 2	AGCTTCTTCACACGCTACCC	GTGGCTGAGCAGAGAGTTCC
GADD45 G	growth arrest and DNA damage inducible gamma	GACACAGTTCCGGAAAGCAC	CCACGTTCAAGACTTTGGCTG

GDPD1	glycerophosphodiester phosphodiesterase domain containing 1	TTTTGAGGCCTTTCCTAACACTCC	CGATTATACCGCTTCACCAACTC
GLUL	glutamate-ammonia ligase	GGTTCTGGCGATGGTTGAGAC	GGGACATGTACACCTGCTTG
GRASP	trafficking regulator and scaffold protein tamalin	ATGAGAAGTGGGGAGAGTACAG G	GACTCCAGCGTGTCGTAGATG
H6PD	hexose-6-phosphate dehydrogenase/glucose 1-dehydrogenase	AGTCTTGCTGAGCGCAAGG	CCCGTCACTCCGACAAGTG
HERPUD 1	homocysteine inducible ER protein with ubiquitin like domain 1	CTGCTCCAGCCCCTATTCAC	CACCCAACGTGATGCCTTG
HNRNPL L	heterogeneous nuclear ribonucleoprotein L like	TGGCTCGGTAATTGAGAGGAG	GCCTCTTCTGCGAGGGTC
IL6R	interleukin 6 receptor	GAGTGGGAAGTCGCACTGAC	CGGCTCTCTACACACACTGC
ITGA5	integrin subunit alpha 5	TCGGGGGCTTCAACTTAGAC	CACACTGACCCCGTCTGTTC
KANK1	KN motif and ankyrin repeat domains 1	TGCATGACTCCTCACTCCTTTC	GGCACTGCCGTTAACCTTTG
KCNAB1	potassium voltage-gated channel subfamily A member regulatory	GCAGGGAAAGACAAATCTCCCAA G	CACCCATGTTCCAAGACCCAAG
KLF13	Kruppel like factor 13	CCCTAGGGTCCAAGCTCTTTC	GCACAGGATCGGAAGGTCTG
KLF9	Kruppel like factor 9	TGGAGGGGGTTTGGTTTGTG	ACCGTTCCGGCATTCTCTTG
LOX	lysyl oxidase	GGCGAAGGGTGAGGAGTAAG	GACGCCTGGATGTAGTAGGG
LPIN1	lipin 1	AAGGAGAATCCACCAGGAGAC	CTGTTAAGCAGGTGGCCTTG
MEGF9	multiple EGF like domains 9	GCAGATGTTTCGGGATTGTTGG	ATGTATGGGCGTCGTCAGGG
MGLL	monoglyceride lipase	CGCAAACGAGGATCCGCTG	GCTGGAAGGGTCTTCAGGTC
MKNK2	MAPK interacting serine/threonine kinase 2	CTCCATCCTGAGCCACATCCAC	TGTGGGCGATGCCTTTGTTATG
MTHFD2	methylenetetrahydrofolate dehydrogenase (NADP+ dependent)	CTCCAGGCCAGCTCAAGAAG	GGTGAGGTACTGCAGAGGTATG
MXD4	MAX dimerization protein 4	TCAGGGGTCATCTGGCAAAC	GTGCAGACAGGCAGGACTAC
NEK7	NIMA related kinase 7	CCAACTTCCTGAGTTCTAAAGTTC	AGCCCATATCCGGTCGTAAG
NFIL3	nuclear factor, interleukin 3 regulated	AACCCGCAGTGCTCAGTC	GAGGCGCTTTTTGTCTTCCTG
NFKBIA	NFKB inhibitor alpha	TGCTCAGGAGCCCTGTAATG	GCCCCACACTTCAACAGGAG
NPM3	nucleophosmin/nucleoplas min 3	CGGCACCAGATTGTTACGATG	ATGGGGCACAGCTCAACTTC
PER1	period circadian regulator 1	GAGGTTGGTGGACCATGGAG	CACAAATGCCATCGGCAGAG
PER2	period circadian regulator 2	GACATGAGACCAACGAAAACTGC	AGGCTAAAGGTATCTGGACTCTG
PIK3R1	phosphoinositide-3-kinase regulatory subunit 1	AAGTGGCACTGCCTAAGAAC	CTCCCCCTTCCCAAAGCTAAC
PLD1	phospholipase D1	CTGAGCTCCAGCTGTGCCAGA	GTATTTACCCGTGGCTCGTTTTTC
PLEKHF1	pleckstrin homology and FYVE domain containing 1	ACTGCGGTGTGGACTCG	AAGTGGTCCACCATCGTCTC
PPP1R3C	protein phosphatase 1 regulatory subunit 3C	GCGTTGTGTTTGCTGACTCC	AGATCCCACGCTGGTTCTTC
RASA3	RAS p21 protein activator 3	GATCAAGATCGGTGAAGCCAAAA AC	GTTCACCGTGCAGTAGCAATC
RGS2	regulator of G protein signaling 2	TGCAGACCCATGGACAAGAG	GCTCAAACGGGTCTTCCAATC

RHOB	ras homolog family member B	AACTGACTTGGGGAGGACAC	GGTAGCTGGTGGAGGGTTC
RHOJ	ras homolog family member J	TGCTCGGACTGTATGACACC	CACATCCGTGTTGGGGTAGG
RHOU	ras homolog family member U	GGGAGCACCTGTCTGGATTG	ACACTTGCTGTTGGCCTTTC
SDC4	syndecan 4	GCCGAGTCGATCCGAGAG	CCCCACTACATCCTCATCGTC
SELENOP	selenoprotein P	GAGCTGCCAGAGTAAAGCAAAG	CACATATTGCAAGTAGACAACCA C
SESN1	sestrin 1	GGCCACACATTCAGACCTCC	GACCGGCATCTCATCCACAC
SGK1	serum/glucocorticoid regulated kinase 1	TAGCAGGGAGGCTTATTCCAG	TGCCCAAGGATATGCAGGTTG
SLC10A6	solute carrier family 10 member 6	ATTACCCTTGTGTGCCTGACC	ACCAACAACGGCCCCAATC
SLC19A2	solute carrier family 19 member 2	TGACCGAGAGGGAGGAACC	GGCGAGAGGAGTAGCACATC
SLC45A3	solute carrier family 45 member 3	CCCGGAGACACTATGATGAAGG	ACCAGAGAGAAGACCAGGGAG
SMOX	spermine oxidase	CGCTCGCCGCAGACTTAC	AGAGGGTCATCCGCACTGTC
SNTA1	syntrophin alpha 1	GGCTCGGAGGCGAAGATG	CACGGTCAGCACGTCCTC
SPHK1	sphingosine kinase 1	GGGATTGTAGGCTTAGTCACACG	GGGAAGTCCAGAGAGCTGAGG
SPSB1	splA/ryanodine receptor domain and SOCS box containing 1	GGCGAACATGGGTCAGAAGG	GCTGGACATCATAGGACACAGG
STOM	stomatin	TGGATTTTGGTGGCGTTCTC	GGCTGCAATAACCTTTATGCACA TC
TBL1XR1	TBL1X receptor 1	CACTGCCAATGTGGGATTACAG	TCCCACTTAAACCATGAGGTC
TGFBR3	transforming growth factor beta receptor 3	TTTCCTCTTCCCAGCGAGTG	GCGGCAAAACTACGCCATC
THBD	thrombomodulin	TAACGAAGACACAGACTGCGATT	CTAGCCCACGAGGTCAAGGT
TNFAIP3	TNF alpha induced protein 3	CGCCAAGAGAGATCACACCC	GCGATCCTTTCGCAAAGTCC
TOB2	transducer of ERBB2, 2	TTGGCATCAGGGTCCCAATC	TACTTGGGGTCCCTGGGTAG
TP53INP1	tumor protein p53 inducible nuclear protein 1	CGTCTGGGTACCTGAACGAG	TGCACAGGGTGCTTATTCAAC
TPST2	tyrosylprotein sulfotransferase 2	GCGTCTCGCCACGATG	GGTATTCCACGTGGTTGGTG
TSC22D3	TSC22 domain family member 3	CCTCGAGTCACTTCCCTTCAAC	GCGAAGGCTGCAGAACGAAC
UBE2G2	ubiquitin conjugating enzyme E2 G2	GGCCGAGTACAAACAATTAACAC	CACCAAACTCAAAGCAGGTGTC
USP2	ubiquitin specific peptidase 2	CTTCGTCAGCTGGTGCTCACTG	TGTAGCGGGCCGATTCTGTG
USP54	ubiquitin specific peptidase 54	AAGGGACCAGGCTACATTGG	TTGTCCCATCCAGATCTTCAGC
VDR	vitamin D receptor	CCCAGCTGGACGGAGAAATG	GAGGATTGAGGGAGGCAAGC
VLDLR	very low density lipoprotein receptor	CAGCCGATGGAAGTGTGATG	TCCAGGACACTGGGATACAC
XDH	xanthine dehydrogenase	GCACTAACACTGTGCCCAAC	TGGTCTGACAAGCCGCATAG
ZFP36	ZFP36 ring finger protein	CTCTCGCTGCCACTTCATCC	AGCTGATGCTCTGGCGAAG
ZHX3	zinc fingers and homeoboxes 3	TAGCACCCTGAATCTTGGGAC	AGTTGCATTTCAGTCACGCC

GAPDH	glyceraldehyde-3-phosphate dehydrogenase	ACACCCACTCCTCCACCTTTG	TCTCTCTTCCTCTTGTGCTCTTG
HPRT1	hypoxanthine phosphoribosyltransferase 1	GGATTTGGAAAGGGTGTTTATTCC	CCTCCCATCTCCTTCATCACATC
TBP	TATA-Box binding protein	TGAGAAGAGTGTGCTGGAGATG	CGTAAGGTGGCAGGCTGTTG
ACTB	actin beta	CTATCCAGGCTGTGCTATCC	CTTAATGTCACGCACGATTTCC
RPLP0	ribosomal protein lateral stalk subunit P0	GGCGACCTGGAAGTCCAACT	CCATCAGCACCACAGCCTTC

Supplementary Table S2. Sequences of small interfering RNAs (siRNAs) targeting 17 glucocorticoid receptor (GR) target genes and non-targeting siRNA.

Gene name	siRNA sequence (Sense [S], antisense [A])	
ARID5B	#1	S: CUGUGAAACUGUCCUACAU<br>A: AUGUAGGACAGUUUCACAG
	#2	S: UGAGCAUUGUACUUACCUU<br>A: AAGGUAAGUACAAUGCUCA
BCL2L1	#1	S: GGAGAACCACUACAUGCAG<br>A: CUGCAUGUAGUGGUUCUCC
	#2	S: CUCUGAUAUGCUGUCCCUG<br>A: CAGGGACAGCAUAUCAGAG
BCL6	#1	S: CACAGUGACAAACCCUACA<br>A: UGUAGGGUUUGUCACUGUG
	#2	S: GAGCAAAAUUUUGGACUGU<br>A: ACAGUCCAAAAUUUUGCUC
CAT	#1	S: CACUGAUUUCACAACAGAU<br>A: AUCUGUUGUGAAAUCAGUG
	#2	S: CUAUGUGAACGUGCUGAAU<br>A: AUUCAGCACGUUCACAUAG
DDIT4	#1	S: GUAUUGUCACUCCCAUGUU<br>A: AACAUGGGAGUGACAAUAC
	#2	S: CGAGUCUGGCAUUCUGCUU<br>A: AAGCAGAAUGCCAGACUCG
DUSP1	#1	S: CUCAGUGUGUGACUUGGUU<br>A: AACCGGAUCACACACUGAG
	#2	S: CUCAGUCCAAAAGCGGCUU<br>A: AAGCCGCUUUUGGACUGAG
FKBP5	#1	S: CUGUUGGGAAAGCUCUGUA<br>A: UACAGAGCUUUCCCAACAG
	#2	S: GAGAGAUAUGCCAUUUACU<br>A: AGUAAAUGGCAUAUCUCUC
GLUL	#1	S: GACGUACUCUGGUUAGGUU<br>A: AACCUAACCAGAGUACGUC
	#2	S: GACCUGACGUACUCUGGUU<br>A: AACCAGAGUACGUCAGGUC
KLF13	#1	S: CACCAAAUUGCACAAUAGA<br>A: UCUAUUGUGCAAUUUGGUG
	#2	S: GAGGAACCCAUAGGACUCA<br>A: UGAGUCCUAUGGGUUCCUC
LOX	#1	S: GUGGAUUGAUAUUACAGAU

		A: AUCUGUAAUAUCAAUCCAC
	#2	S: CUCCUUCCCUCACGUGAUU<br>A: AAUCACGUGAGGGAAGGAG
MTHFD2	#1	S: GUAUGUUACCCUUCAGUAA<br>A: UUACUGAAGGGUAACAUAC
	#2	S: GAGGAUGGAUGCUUCACUU<br>A: AAGUGAAGCAUCCAUCCUC
NFIL3	#1	S: GAGAACGCUGGAAACUGAU<br>A: AUCAGUUUCCGACGUUCUC
	#2	S: CACACAAGCUCCGGAUCAA<br>A: UUGAUCCGGAGCUUGUGUG
NFKBIA	#1	S: GUGUUAAGCGUUCAGUGAU<br>A: AUCACUGAACGCUUAACAC
	#2	S: CGAGACUUUCGAGGAAAUA<br>A: UAUUUCCUCGAAAGUCUCG
SLC19A2	#1	S: CACUCUAAUUGUGGUAGAU<br>A: AUCUACCACAAUUAGAGUG
	#2	S: GAACGCUAUGCCCUAGUAU<br>A: AUACUAGGGCAUAGCGUUC
TSC22D3	#1	S: CUGUGUUCAUUUACCAAGU<br>A: ACUUGGUAAAUGAACACAG
	#2	S: GAUGUAUGGUUCCAGGAUU<br>A: AAUCCUGGAACCAUACAUC
ZFP36	#1	S: CGUUACACCAUGGAUCUGA<br>A: UCAGAUCCAUGGUGUAACG
	#2	S: GGUGCUCAAAUUACCCUCC<br>A: GGAGGGUAAUUUGAGCACC
ZHX3	#1	S: GAGACUGCACUAUAUGCUU<br>A: AAGCAUAUAGUGCAGUCUC
	#2	S: AGUGAUGACCGGCUACGUU<br>A: AACGUAGCCGGUCAUCACU
Non-targeting siRNA (NT)	S: GAACUGAUGACAGGGAGGC<br>A: GCCUCCCUGUCAUCAGUUC	

Supplementary Table S3. Fragments per kilobase exon per million fragments mapped (FPKM) values of 108 glucocorticoid receptor (GR) target genes in human dermal fibroblasts.

Gene ID	Gene name	Description	FPKM
1490	CTGF	connective tissue growth factor	7.99939
444	ASPH	aspartate beta-hydroxylase	7.90258
1026	CDKN1A	cyclin dependent kinase inhibitor 1A	7.85427
4015	LOX	lysyl oxidase	7.74251
140609	NEK7	NIMA related kinase 7	7.60868
3678	ITGA5	integrin subunit alpha 5	7.29208
1843	DUSP1	dual specificity phosphatase 1	6.77697
133	ADM	adrenomedullin	6.64634
2040	STOM	stomatin	6.45502
8877	SPHK1	sphingosine kinase 1	6.09284
388	RHOB	ras homolog family member B	5.99749
8459	TPST2	tyrosylprotein sulfotransferase 2	5.94972
11343	MGLL	monoglyceride lipase	5.87040
11072	DUSP14	dual specificity phosphatase 14	5.85348
10797	MTHFD2	methylenetetrahydrofolate dehydrogenase (NADP+ dependent) 2, methenyltetrahydrofolate cyclohydrolase	5.81478
6385	SDC4	syndecan 4	5.76135
2355	FOSL2	FOS like 2, AP-1 transcription factor subunit	5.09847
9709	HERPUD1	homocysteine inducible ER protein with ubiquitin like domain 1	4.93737
10360	NPM3	nucleophosmin/nucleoplasmin 3	4.85651
598	BCL2L1	BCL2 like 1	4.68294
2752	GLUL	glutamate-ammonia ligase	4.56054
7327	UBE2G2	ubiquitin conjugating enzyme E2 G2	4.54276
30845	EHD3	EH domain containing 3	4.40184
92906	HNRNPLL	heterogeneous nuclear ribonucleoprotein L like	4.39435
847	CAT	catalase	4.37573
6446	SGK1	serum/glucocorticoid regulated kinase 1	4.35103
22821	RASA3	RAS p21 protein activator 3	4.29961
2872	MKNK2	MAPK interacting serine/threonine kinase 2	4.24330
137964	AGPAT6	glycerol-3-phosphate acyltransferase 4	4.14420
4792	NFKBIA	NFKB inhibitor alpha	4.06086
2289	FKBP5	FK506 binding protein 5	4.05208
79718	TBL1XR1	TBL1X receptor 1	4.04971
10766	TOB2	transducer of ERBB2, 2	4.00929
23189	KANK1	KN motif and ankyrin repeat domains 1	3.88590
10608	MXD4	MAX dimerization protein 4	3.62073

84159	ARID5B	AT-rich interaction domain 5B	3.59740
7538	ZFP36	ZFP36 ring finger protein	3.43684
9563	H6PD	hexose-6-phosphate dehydrogenase/glucose 1-dehydrogenase	3.41250
4783	NFIL3	nuclear factor, interleukin 3 regulated	3.41038
23051	ZHX3	zinc fingers and homeoboxes 3	3.39646
6640	SNTA1	syntrophin alpha 1	3.32358
4299	AFF1	AF4/FMR2 family member 1	3.27824
54541	DDIT4	DNA damage inducible transcript 4	3.04828
5295	PIK3R1	phosphoinositide-3-kinase regulatory subunit 1	3.04164
168667	BMPER	BMP binding endothelial regulato	3.01491
51621	KLF13	Kruppel like factor 13	2.97568
5507	PPP1R3C	protein phosphatase 1 regulatory subunit 3C	2.94475
57381	RHOJ	ras homolog family member J	2.86835
23175	LPIN1	lipin 1	2.80694
604	BCL6	BCL6 transcription repressor	2.73053
27244	SESN1	sestrin 1	2.72433
7436	VLDLR	very low density lipoprotein receptor	2.69124
80020	FOXRED2	FAD dependent oxidoreductase domain containing 2	2.63053
1831	TSC22D3	TSC22 domain family member 3	2.57099
54498	SMOX	spermine oxidase	2.55313
7421	VDR	vitamin D receptor	2.44356
55664	CDC37L1	cell division cycle 37 like 1	2.43365
80176	SPSB1	splA/ryanodine receptor domain and SOCS box containing 1	2.18838
272	AMPD3	adenosine monophosphate deaminase 3	2.13536
94241	TP53INP1	tumor protein p53 inducible nuclear protein 1	1.99646
5997	RGS2	regulator of G protein signaling 2	1.99409
91768	CABLES1	Cdk5 and Abl enzyme substrate 1	1.96144
7049	TGFBR3	transforming growth factor beta receptor 3	1.91585
7128	TNFAIP3	TNF alpha induced protein 3	1.91042
81617	CAB39L	calcium binding protein 39 like	1.87626
687	KLF9	Kruppel like factor 9	1.84417
1955	MEGF9	multiple EGF like domains 9	1.82488
159195	USP54	ubiquitin specific peptidase 54	1.73847
104	ADARB1	adenosine deaminase RNA specific B1	1.44429
79156	PLEKHF1	pleckstrin homology and FYVE domain containing 1	1.38096
10560	SLC19A2	solute carrier family 19 member 2	1.21869
51129	ANGPTL4	angiopoietin like 4	1.16985
50650	ARHGEF3	Rho guanine nucleotide exchange factor 3	1.14538
85414	SLC45A3	solute carrier family 45 member 3	1.03253

3570	IL6R	interleukin 6 receptor	0.96553
154	ADRB2	adrenoceptor beta 2	0.90602
5187	PER1	period circadian regulator 1	0.75720
6414	SELENOP	selenoprotein P	0.74143
7881	KCNAB1	potassium voltage-gated channel subfamily A member regulatory beta subunit 1	0.73519
7498	XDH	xanthine dehydrogenase	0.67309
10912	GADD45G	growth arrest and DNA damage inducible gamma	0.63021
284161	GDPD1	glycerophosphodiester phosphodiesterase domain containing 1	0.56887
160622	GRASP	trafficking regulator and scaffold protein tamalin	0.56162
5337	PLD1	phospholipase D1	0.51654
8864	PER2	period circadian regulator 2	0.43198
9099	USP2	ubiquitin specific peptidase 2	0.35694
58480	RHOU	ras homolog family member U	0.35330
345274	SLC10A6	solute carrier family 10 member 6	0.14802
7056	THBD	thrombomodulin	0.10163
183	AGT	angiotensinogen	0.08356
10461	MERTK	MER proto-oncogene, tyrosine kinase	0.05754
6337	SCNN1A	sodium channel epithelial 1 subunit alpha	0.04604
729359	PLIN4	perilipin 4	0.04494
29887	SNX10	sorting nexin 10	0.03995
9844	ELMO1	engulfment and cell motility 1	0.03528
79092	CARD14	caspase recruitment domain family member 14	0.02974
5166	PDK4	pyruvate dehydrogenase kinase 4	0.02687
79170	PRR15L	proline rich 15 like	0.00322
7704	ZBTB16	zinc finger and BTB domain containing 16	0.00318
3934	LCN2	lipocalin 2	0.00192
3768	KCNJ12	potassium inwardly rectifying channel subfamily J member 12	0.00174
64344	HIF3A	hypoxia inducible factor 3 subunit alpha	0.00047
339	APOBEC1	apolipoprotein B mRNA editing enzyme catalytic subunit 1	0
54769	DIRAS2	DIRAS family GTPase 2	0
3484	IGFBP1	insulin like growth factor binding protein 1	0
56246	MRAP	melanocortin 2 receptor accessory protein	0
69068	TCIM(1810011O10RIK)	transcriptional and immune response regulator	0
100040353	2810416G20RIK	RIKEN cDNA 2810416G20 gene	0
