# Supplementary figures and images for: Identification of Glucocorticoid Receptor Target Genes That Potentially Inhibit Collagen Synthesis in Human Dermal Fibroblasts
Source: Biomolecules. 2023 Jun 11;13(6):978. doi: 10.3390/biom13060978 (PMC10296022; doi:10.3390/biom13060978)

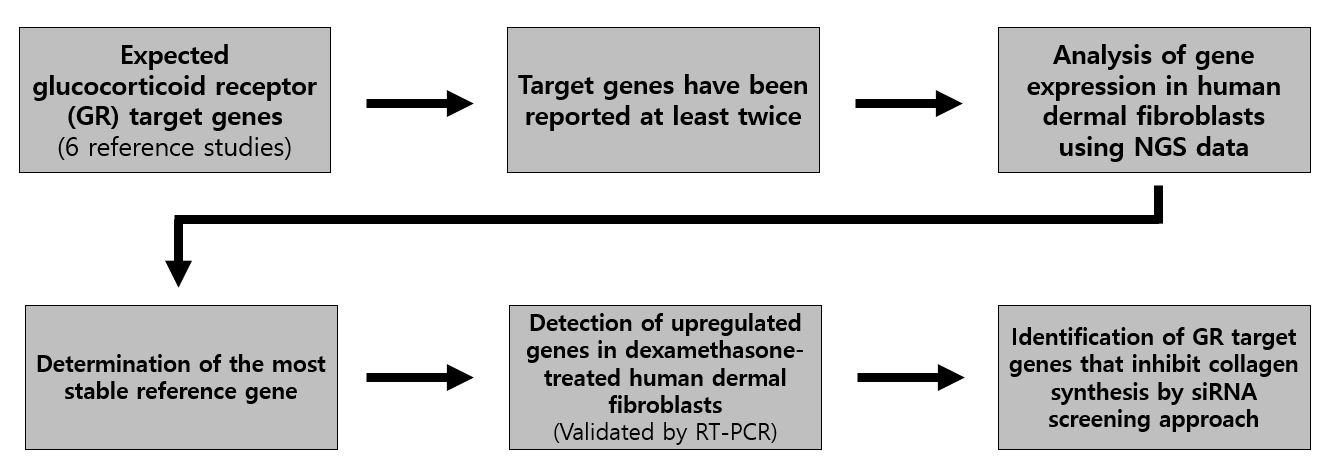

Supplement: Supplementary file 1 [file biomolecules-13-00978-s001.zip › Supplementary figure 1..JPG]

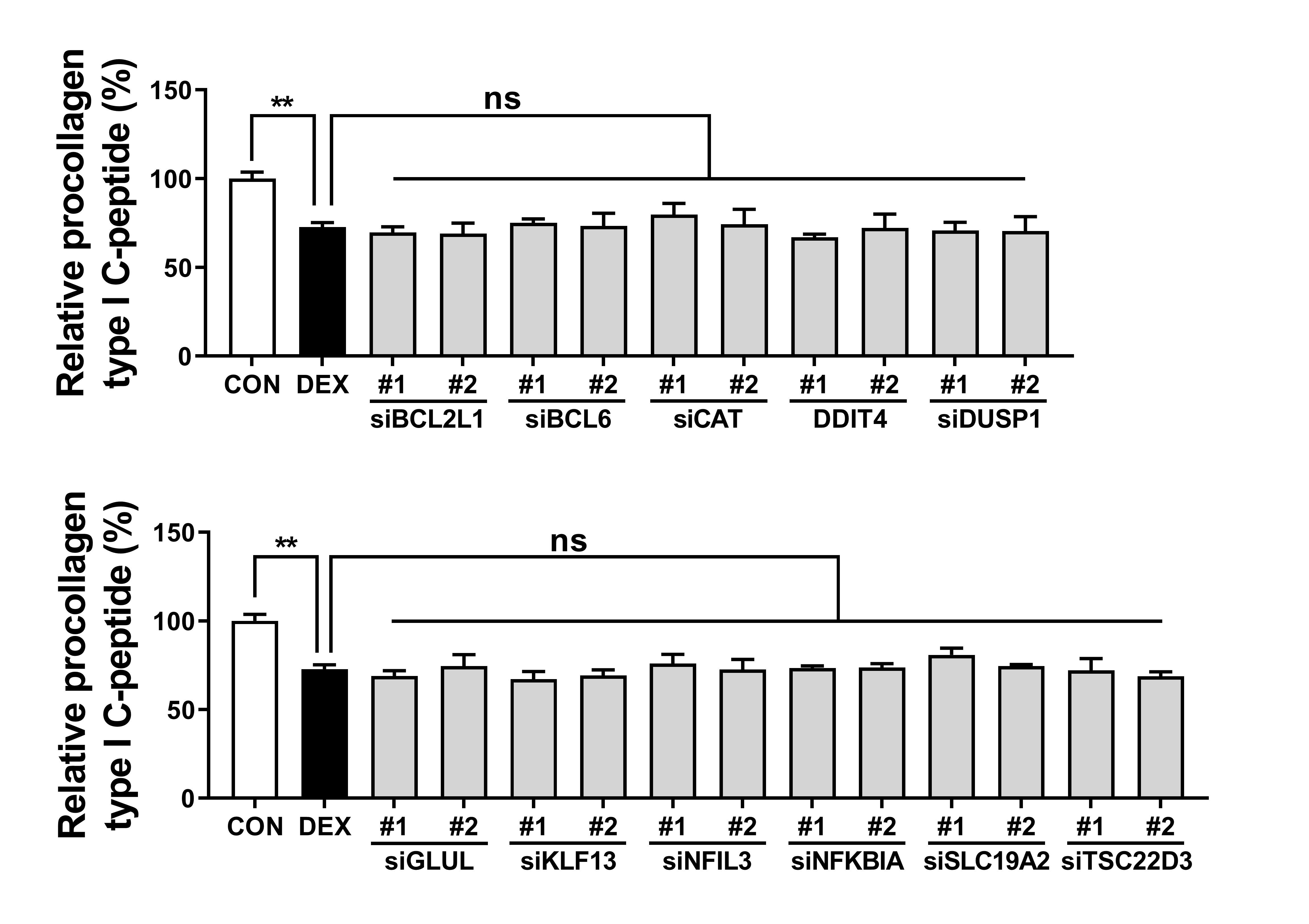

Supplement: Supplementary file 1 [file biomolecules-13-00978-s001.zip › Supplementary figure 2..tif]

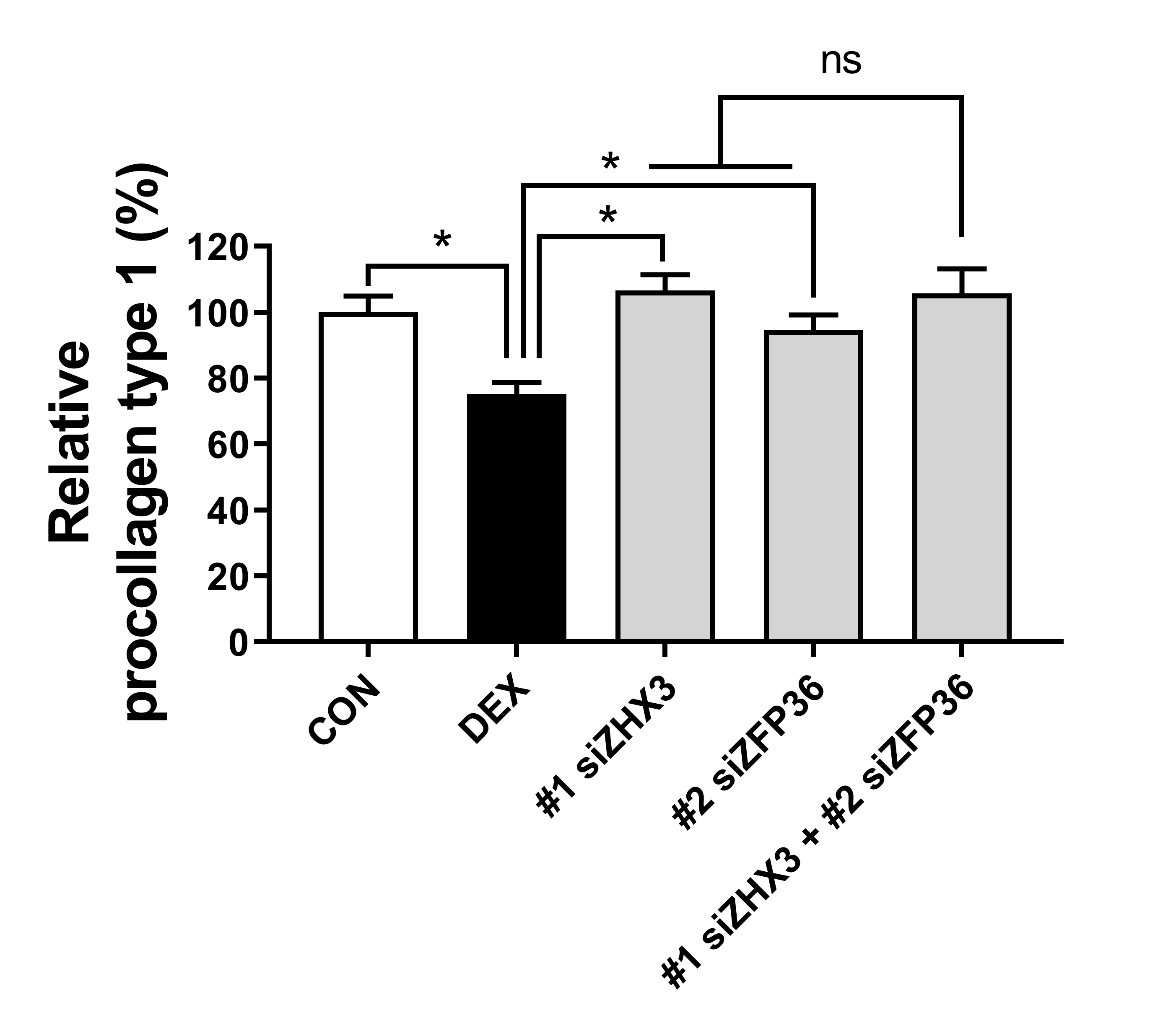

Supplement: Supplementary file 1 [file biomolecules-13-00978-s001.zip › Supplementary figure 3.tif]
